# Supplementary material for: Gibberellin Signaling Repressor LlDELLA1 Controls the Flower and Pod Development of Yellow Lupine (Lupinus luteus L.)
Source: Int J Mol Sci. 2020 Mar 6;21(5):1815. doi: 10.3390/ijms21051815 (PMC7084671; doi:10.3390/ijms21051815)
Supplement: Supplementary file 1 [file ijms-21-01815-s001.pdf]

```
     1-62  at agt act atc tgc agt agt gac aaa aac ccc aaa cca caa atc act ccc tcc aca cac tta
   63-122     ccc tct aac ctt gtg ttt ttt gag cat cac ttc tat tct cta atc act atg aag aga gag
                                                                                M   K   R   E      1-4
  123-182     cat cac cat ctt cat ccc aat ccc agt gac tca tca tct tcc atg tca gct gct acc acc
               H   H   H   L   H   P   N   P   S   D   S   S   S   S   M   S   A   A   T   T      5-24
  183-242     gga aaa cca aac cta tgg gag gaa gac atc ggc ggc ggc ggc ggc ggc ggc ggc atg gat
               G   K   P   N   L   W   E   E   D   I   G   G   G   G   G   G   G   G   M   D      25-44
  243-302     gag ctt ctc gcg gtg gtg gga tac aaa gtt agg tca caa gac atg gcg gag gta gct cag
               E   L   L   A   V   V   G   Y   K   V   R   S   Q   D   M   A   E   V   A   Q      45-64
  303-362     aaa ata gaa caa ctt gaa gaa gct atg aca agt gtt gaa act aac att tct tca ctc tct
               K   I   E   Q   L   E   E   A   M   T   S   V   E   T   N   I   S   S   L   S      65-84
  363-422     tcc aac acc gtc cat tac aac cca tca gat atc tcc acg tgg ctt gaa tcc atg att tct
               S   N   T   V   H   Y   N   P   S   D   I   S   T   W   L   E   S   M   I   S      85-104
  423-482     ggc ctc agc cca tca caa cac caa gtt caa gac gat tcc ttc ttt act tca act gaa tcc
               G   L   S   P   S   Q   H   Q   V   Q   D   D   S   F   F   T   S   T   E   S      105-124
  483-542     tca acc atc act tcc tct tct gat tat gac ctc gag gct atc cct ggt aaa gca att tac
               S   T   I   T   S   S   S   D   Y   D   L   E   A   I   P   G   K   A   I   Y      125-144
  543-602     act aac atc aat aat aac aac att gac aaa caa acg aag cgg atg aga act tct ttg tta
               T   N   I   N   N   N   N   I   D   K   Q   T   K   R   M   R   T   S   L   L      145-164
  603-662     tta ccc tcc tct tcg agt cgg cca gtt gta ttg gcc gac tcg caa gag aat gga att aga
               L   P   S   S   S   S   R   P   V   V   L   A   D   S   Q   E   N   G   I   R      165-184
  663-722     tta gtc cat acc tta ttg gct tgc gcc gag gcc gta agc caa act aat ctg tct att gct
               L   V   H   T   L   L   A   C   A   E   A   V   S   Q   T   N   L   S   I   A      185-204
  723-782     gaa gcc ttg ctt cac cag atc ggg ttt ctt gct ttg tct caa cct ggc gcg atg cgt aag
               E   A   L   L   H   Q   I   G   F   L   A   L   S   Q   P   G   A   M   R   K      205-224
  783-842     gtt gcg acg tat ttc gct gaa gct ctg gct cgg cga gtc tac ggg ctc tac ccg gaa acg
               V   A   T   Y   F   A   E   A   L   A   R   R   V   Y   G   L   Y   P   E   T      225-244
  843-902     ccg ttg ctc aac caa cat aac aac cac cac cac ctc tcc gag atg ctt cag atc cat ttc
               P   L   L   N   Q   H   N   N   H   H   H   L   S   E   M   L   Q   I   H   F      245-264
  903-962     tat gaa acg tgt cct tac ctc aaa ttt gca cac ttc act gcg aat caa gct ata ctc gaa
               Y   E   T   C   P   Y   L   K   F   A   H   F   T   A   N   Q   A   I   L   E      265-284
 963-1022     gct ttt caa ggg aag gat cgt gtt cat gta atc gat ttc agt atg aac caa ggg atg cag
               A   F   Q   G   K   D   R   V   H   V   I   D   F   S   M   N   Q   G   M   Q      285-304
1023-1082     tgg ccc gca ctg ttg caa gca ctt gcg ctt cgt ccc ggt ggt cct ccg gcg ttc agg ctt
               W   P   A   L   L   Q   A   L   A   L   R   P   G   G   P   P   A   F   R   L      305-324
1083-1142     acc gga atc gga cct ccg gcg atg gac aac tcc gat cat ttg caa gaa gtt ggt tgg aaa
               T   G   I   G   P   P   A   M   D   N   S   D   H   L   Q   E   V   G   W   K      325-344
1143-1202     ctc gct cag ctt gcg gaa acg att cac gtc gag ttt gaa ttt cgt gga ttc gtg gct aat
               L   A   Q   L   A   E   T   I   H   V   E   F   E   F   R   G   F   V   A   N      345-364
1203-1262     agt ctc gcg gat ctg gac gcg tcg atg ctt gaa ctc agc ccg agt gag acc gag tca gtg
               S   L   A   D   L   D   A   S   M   L   E   L   S   P   S   E   T   E   S   V      365-384
1263-1322     gcg gtt aac tca gta ttc gag ttt cat aag ctc tta gct cgt cct ggt gcg gtg gag aag
               A   V   N   S   V   F   E   F   H   K   L   L   A   R   P   G   A   V   E   K      385-404
1323-1382     att ctc tct gta gta aaa cag gtt aaa ccg gaa atc gtc aca gtt gtt gaa cta gaa gcg
               I   L   S   V   V   K   Q   V   K   P   E   I   V   T   V   V   E   L   E   A      405-424
1383-1442     aac cat aac gga ccg ggt ttt ctg gac cgg ttc act gag tca ctg cat tat tac tca aca
               N   H   N   G   P   G   F   L   D   R   F   T   E   S   L   H   Y   Y   S   T      425-444
1443-1502     atg ttt gac tcg ctt gaa gga tca gca cca gtg gag tca caa gat aag tta ttg tca gag
               M   F   D   S   L   E   G   S   A   P   V   E   S   Q   D   K   L   L   S   E      445-464
1503-1562     gtt tat tta ggg aag caa atc tgt aac gtg gtg gcg tgt gaa gga ctg gac cgc gtg gaa
               V   Y   L   G   K   Q   I   C   N   V   V   A   C   E   G   L   D   R   V   E      465-484
1563-1622     cgg cac gag acg ttg aac cag tgg aga acc cgg ttc ggt tca gcc ggt ttt att ccg gtt
               R   H   E   T   L   N   Q   W   R   T   R   F   G   S   A   G   F   I   P   V      485-504
1623-1682     cat ttg ggt tca aac gcg ttt aaa caa gcg agt atg tta tta gcg tta ttt gca ggt gga
               H   L   G   S   N   A   F   K   Q   A   S   M   L   L   A   L   F   A   G   G      505-524
1683-1742     gat gga tac aga gtt gaa gag aat aac ggt tgt ttg atg ttg ggt tgg cac aca agg ccg
               D   G   Y   R   V   E   E   N   N   G   C   L   M   L   G   W   H   T   R   P      525-544
1743-1802     tta att gct acc tcc gca tgg aaa ctc ggt gat gta act cag tga ctc aac tca gcg agt
               L   I   A   T   S   A   W   K   L   G   D   V   T   Q   -                          545-558
1803-1862     tga act ttt ttt aat ttt tct gtt ttt aat tta aat ttc aat gga agt ttg atg tgg gta
1863-1922     gtg tat ggt gtg cta tgc tga gct gta tgt tag gcc cac cat tgt cca ttt ctc tgt taa
1923-1982     ttt tcc ttt ttt tta ggt tag gct ctt tat ctt aat ttc ttt ttt tgg ggt tgg gtt aat
1983-2042     ggg ttg gaa tct ctt gta cct ttc agg gtt gga aat caa gaa atg gtt aat gtg tat tta
2043-2067     ttt atg ttt ttc tga caa aaa aaa a
```

Figure S1. Molecular cloning and analyses of *LlDELLA1* in yellow lupine (*Lupinus luteus* L.). The obtained cDNA composed of 2067 bp. Its deduced amino acid sequence contains 558 aa (ExPASy, Translate tool), has a m.w. = 61.321 kD and pI = 5.37 (ExPASy, ProtParam). The positions of nucleotides (black small letters) and amino acids (blue capital letters) are marked on the left and right side, respectively. START and STOP codons (black background) were indicated. UTR regions were marked before ATG (5', 1-110 bp) and after TGA (3', 1788-2067 bp) codons (red small letters).

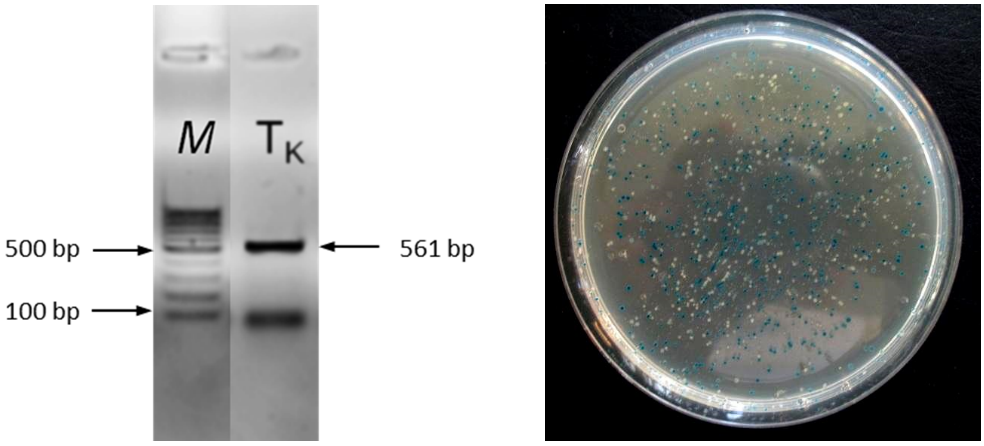

(**A**) (**B**)

Figure S2. Identification of the cDNA fragment (561 bp) of the *LlDELLA1* gene using degenerate primers in yellow lupine var. Taper. (A)Image of electrophoretic separation on a 1% agarose gel in 0.5 × TBE buffer at 5V/cm in the presence of the GeneRuler 100bp DNA Ladder (*M*) (Fermentas, St. Leon-Rot, Germany). (B)An example of a Petri dish with *E. coli* transformed on X-Gal medium. The white bacterial colonies took the recombinant form of the vector, while the blue ones took the vector without insert.

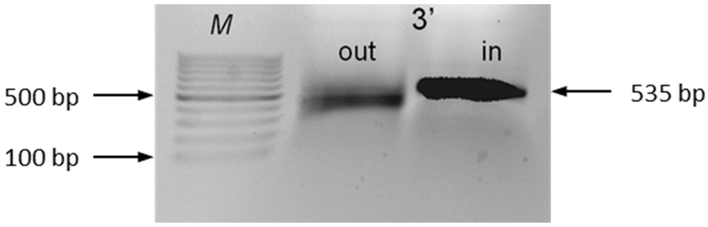

Figure S3. Identification of the 3′ end of the *LlDELLA1* cDNA (535 bp). Image of the electrophoretic separation of the 3′ RACE-PCR reaction product on a 1% agarose gel in 0.5 × TBE buffer at 5V/cm in the presence of the GeneRuler 100bp DNA Ladder (*M*) (Fermentas). OUT—3′ RACE-PCR external reaction; IN—3′ RACE-PCR internal reaction.

Figure S4.

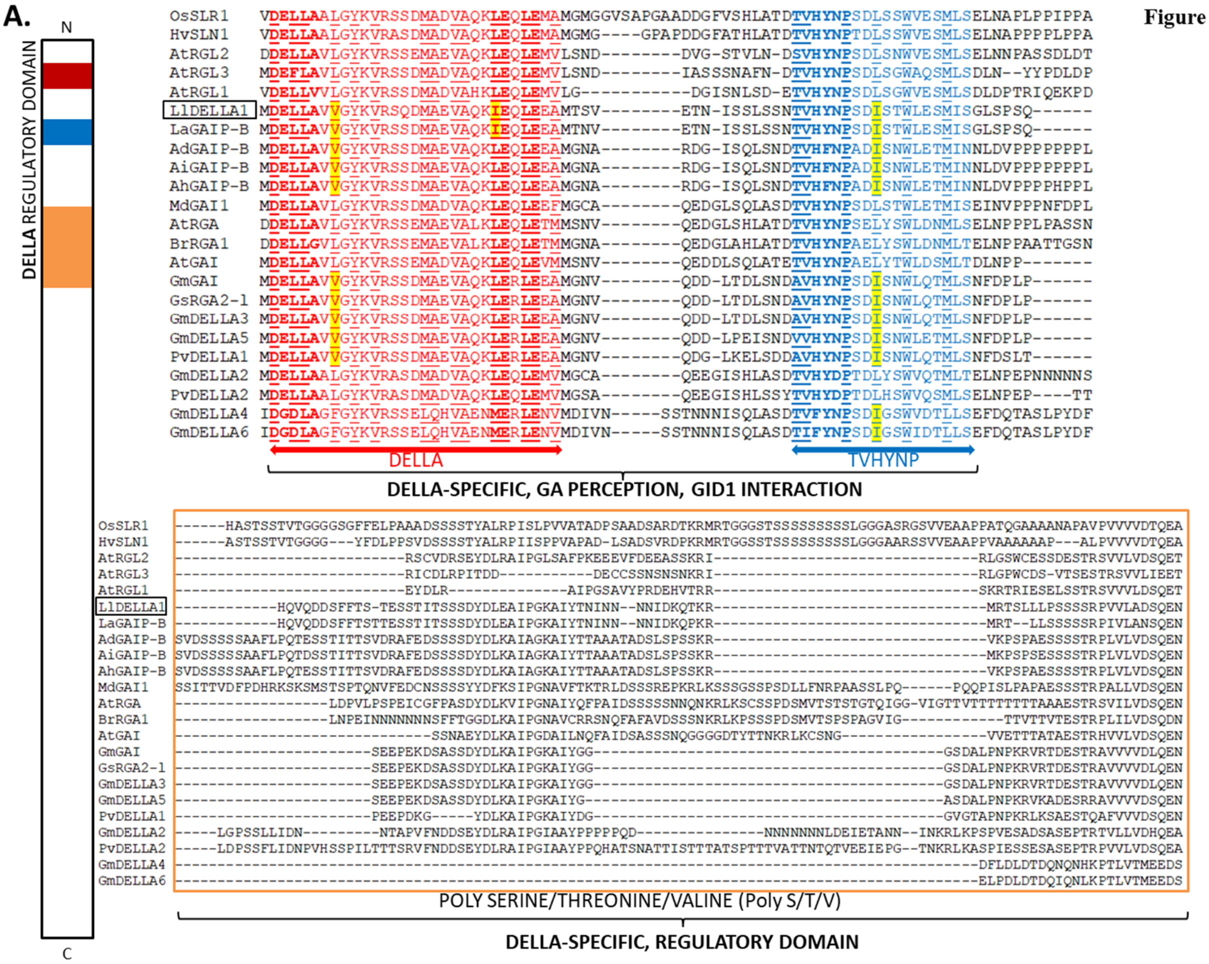
A.
DELLA REGULATORY DOMAIN
N
C
DELLA
TVHYNP
DELLA-SPECIFIC, GA PERCEPTION, GID1 INTERACTION
POLY SERINE/THREONINE/VALINE (Poly S/T/V)
DELLA-SPECIFIC, REGULATORY DOMAIN
OsSLR1
HvSLN1
AtRGL2
AtRGL3
AtRGL1
LlDELLA1
LaGAIP-B
AdGAIP-B
AiGAIP-B
AhGAIP-B
MdGAI1
AtRGA
BrRGA1
AtGAI
GmGAI
GsRGA2-1
GmDELLA3
GmDELLA5
PvDELLA1
GmDELLA2
PvDELLA2
GmDELLA4
GmDELLA6

Figure S4.

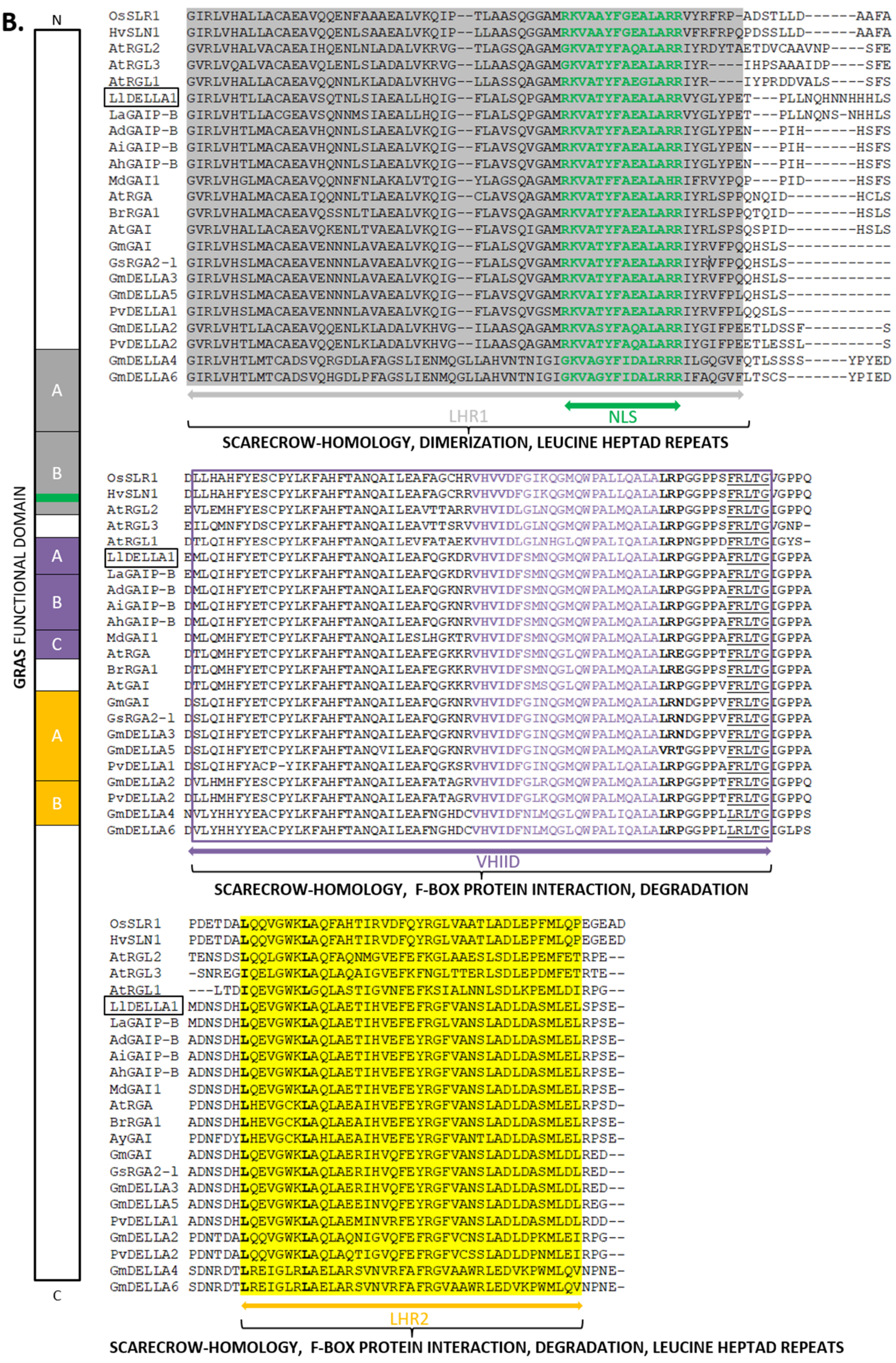
B.
N
C
A
B
A
B
C
A
B
GRAS FUNCTIONAL DOMAIN
LHR1
NLS
SCARECROW-HOMOLOGY, DIMERIZATION, LEUCINE HEPTAD REPEATS
VHIID
SCARECROW-HOMOLOGY, F-BOX PROTEIN INTERACTION, DEGRADATION
LHR2
SCARECROW-HOMOLOGY, F-BOX PROTEIN INTERACTION, DEGRADATION, LEUCINE HEPTAD REPEATS

Figure S4.

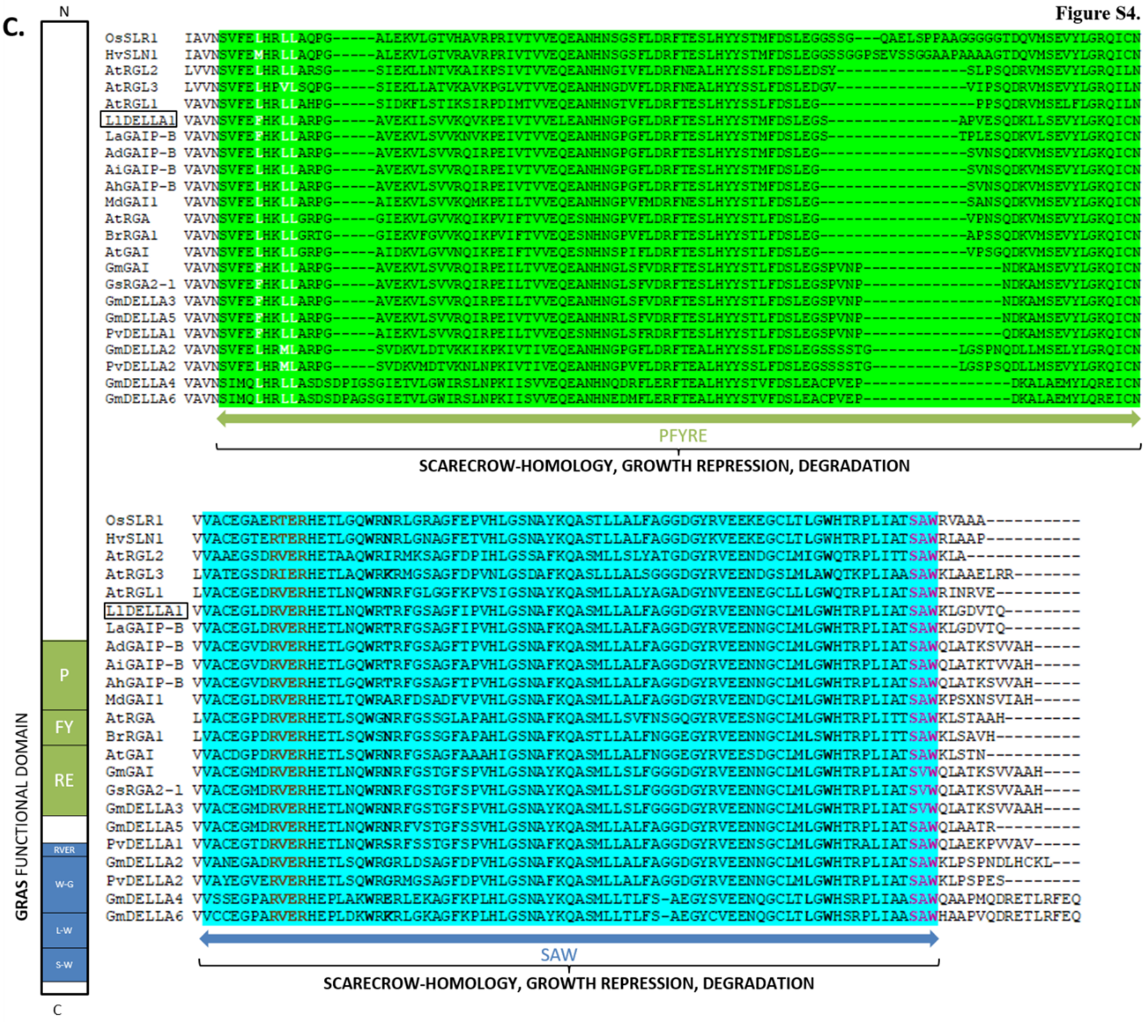

Figure S4. Amino acid sequence alignment of 23 DELLAs from different species. (**A**) Highlighted amino acid residues in the DELLA motif containing <u>D</u>e<u>LL</u>aΦ<u>L</u>x<u>Y</u>x<u>V</u> sequence (red letters), LExLE motif with the consensus sequence <u>MA</u>x<u>VA</u>xx<u>LE</u>x*LE*xΦ (red letters), and in the TVHYNP motif (<u>TV</u>hyn<u>P</u>xx<u>L</u>xx<u>W</u>xxx<u>M</u>) (blue letters) are essential for direct interaction between DELLA and the GID1 surface. 'Φ' represents a non-polar residue, and 'x' can represent any residue. The poly S/T/V motif contains the L(K/R)XI motif likely involved in binding an undetermined GA signaling component. (**B, C**) The GRAS functional domain contains two leucine heptad repeats (LHR1, grey box; LHR2, yellow box), a nuclear localization signal (NLS, green letters), the VHIID (violet box), the PFYRE (green box), LXXLL (white letters), and SAW (blue box) motifs.

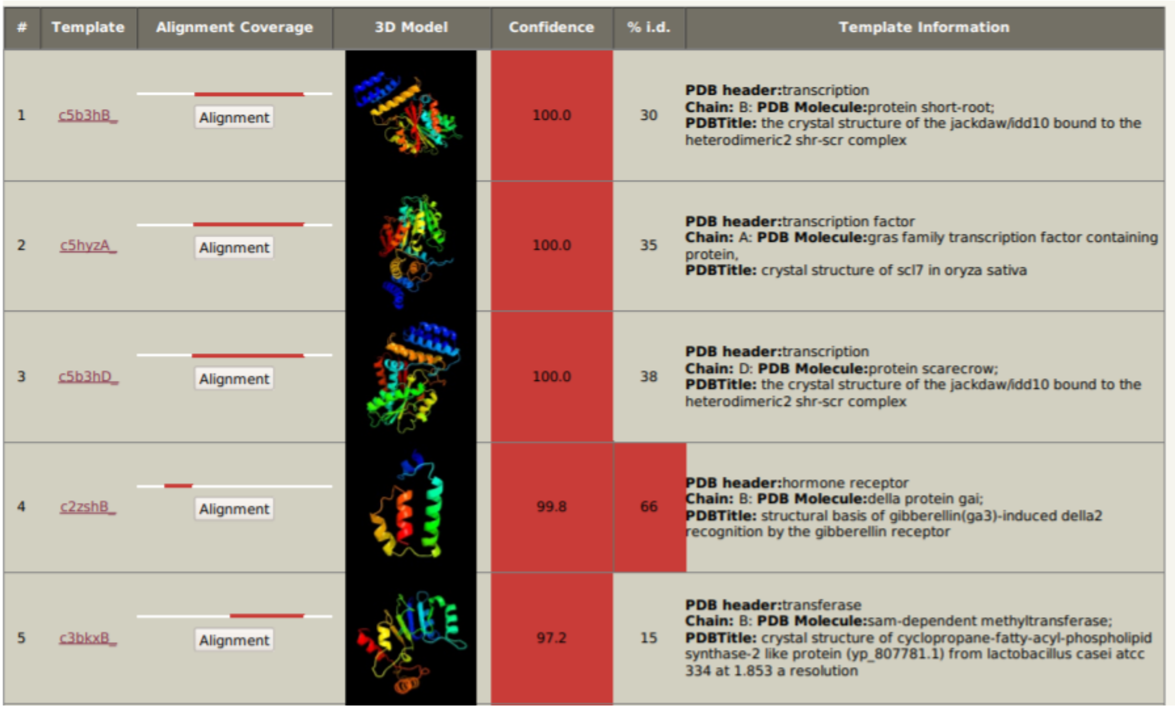

#	Template	Alignment Coverage	3D Model	Confidence	% i.d.	Template Information
1	c5b3hB_	Alignment		100.0	30	**PDB header:**transcription **Chain:** B: **PDB Molecule:**protein short-root; **PDBTitle:** the crystal structure of the jackdaw/idd10 bound to the heterodimeric2 shr-scr complex
2	c5hyzA_	Alignment		100.0	35	**PDB header:**transcription factor **Chain:** A: **PDB Molecule:**gras family transcription factor containing protein, **PDBTitle:** crystal structure of scl7 in oryza sativa
3	c5b3hD_	Alignment		100.0	38	**PDB header:**transcription **Chain:** D: **PDB Molecule:**protein scarecrow; **PDBTitle:** the crystal structure of the jackdaw/idd10 bound to the heterodimeric2 shr-scr complex
4	c2zshB_	Alignment		99.8	66	**PDB header:**hormone receptor **Chain:** B: **PDB Molecule:**della protein gai; **PDBTitle:** structural basis of gibberellin(ga3)-induced della2 recognition by the gibberellin receptor
5	c3bkxB_	Alignment		97.2	15	**PDB header:**transferase **Chain:** B: **PDB Molecule:**sam-dependent methyltransferase; **PDBTitle:** crystal structure of cyclopropane-fatty-acyl-phospholipid synthase-2 like protein (yp_807781.1) from lactobacillus casei atcc 334 at 1.853 a resolution

Figure S5. Top protein models used by phyre^2 to calculate 3D structure of LlDELLA1. The best templates used to calculate LlDELLA1 model were SHORT-ROOT (SHR) from *A. thaliana* (PDB: 5B3H), SCARECROW-LIKE PROTEIN 7 (SCL7) from *O. Sativa* (PDB: 5HYZ), SCARECROW form *A. thaliana* (PDB: c5b3hD), GAI from *A. thaliana* (PDB: c2zshB), S-Adenosyl Methionine-dependent methyltransferase from *Lactobacillus casei* (PDB: c3bkxB). The AtGAI has most identical sequence from PDB templates to LlDELLA1 sequence.

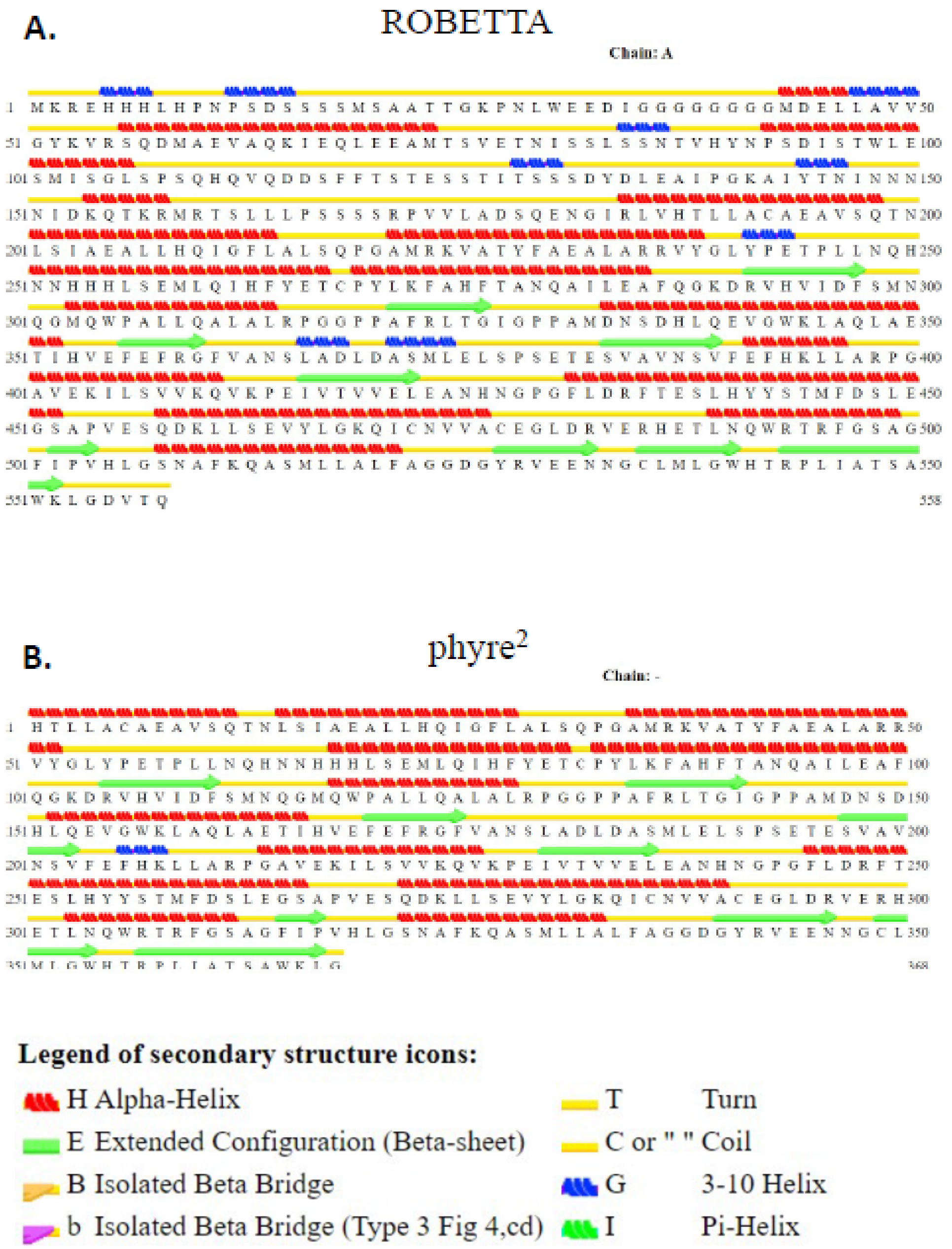

Figure S6. Secondary structure of LlDELLA1 model constructed by ROBETTA (**A**) and phyre^2 (**B**). Alignment visualized by STRIDE web server.

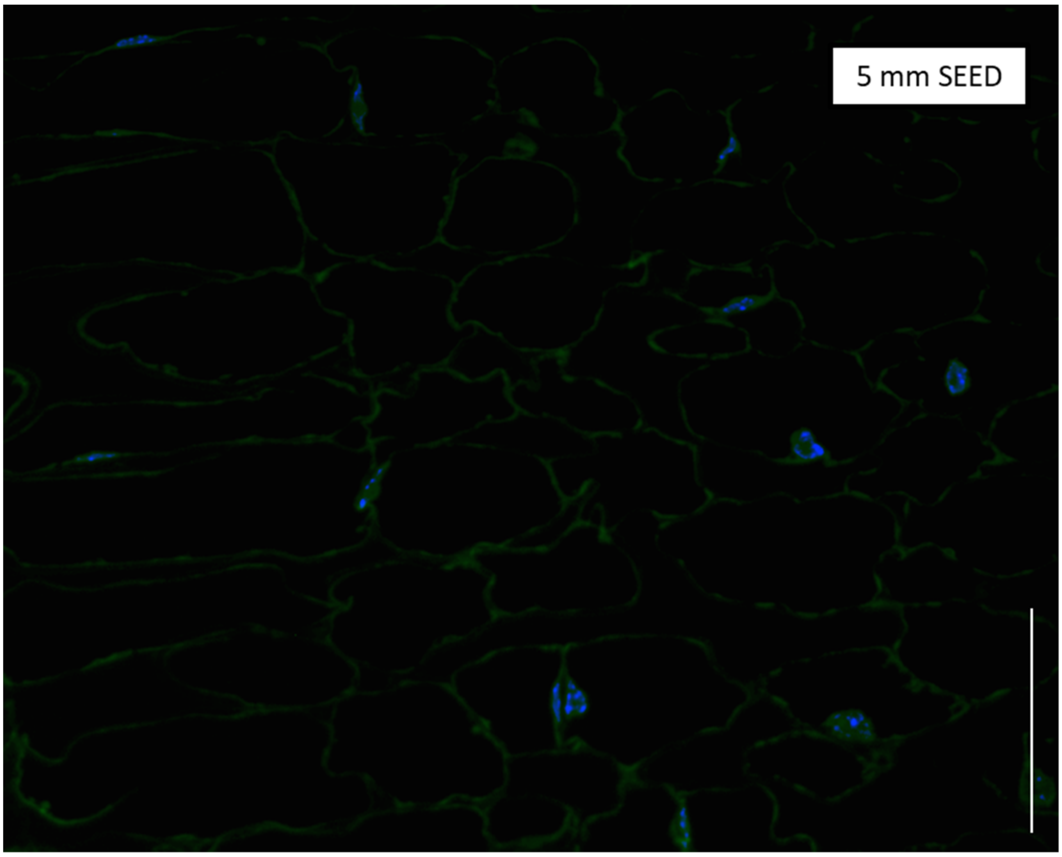

Figure S7. Negative control reaction, required for validation of the immunohistochemical findings, was carried out by omitting the incubation with the primary antibody, and showed no labeling. The autofluorescence signal of the cell walls is visible. DAPI was used to stain cell nuclei. Scale bar = 50 µm.

Table S1. Sequences of degenerate primers designed based on the cDNA sequences of *DELLA* (*GAI, GA INSENSITIVE*) genes derived from closely related species (*Glycine max*, *Phasoleus vulgaris*, *Malus domestica*).

**FP 5'-3' (26 nt, Tm=65,8°C)**	
G. max GAI1	GGGATGCAGTGGCCGGCACTGATGCA
P. vulgaris GAI1	GGGATGCAGTGGCCGGCACTGTTGCA
M. domestica GAI1	GGGATGCAGTGGCCCGCTCTGATGCA
**Degenerate primer**	GGGATGCAGTGGCCSGCWCTGWTGCA
**RP** 5'-3' (28 nt, Tm=65,8-70,2°C)	
G. max GAI1	GACCGCGTGGAAAGGCACGAGACGCTGA
P. vulgaris GAI1	GACCGCGTGGAACGGCACGAGACGCTGA
M. domestica GAI1	GACCGGGTGGAGAGGCACGAGACGTTGA
Degenerate primer	TCARCGTCTCGTGCCKYTCCACSCGGTC

Table S2. Sequences of specific and universal primers used in 3' RACE-PCR. GSIP—gene specific inner primer, GSOP—gene specific outer primer, IP—inner primer, OP—outer primer. .

**Primer sequence (5'-3')**	**Tm (°C)**	
3' GSIP – TGGTGGCGTGTGAAGGACTGGA	74,5	Specific primers
3' GSOP – CAGAGGTTTATTTAGGGAAGCAA	62,5	
3' IP – CGCGGATCCGAATTAATACGACTCACTATAGG	73,7	Universal primers provided by the manufacturer
3' OP – GCGAGCACAGAATTAATACGACT	63,6	

Table S3. Sequences of specific primers and Universal Probe Library (UPL) probes used in qPCR for *LlDELLA1* and *LlACT*. The no. 39 probe for *LlDELLA1* and no 9. probe for *LlACT* were used.

cDNA of gene	Primer sequence (5'-3')	Tm (°C)	%GC	Location of UPL (5'-3 ') relative to primers
LlDELLA1	FP- CGCGTTTAAACAAGCGAGTA<br>RP- CAACCGTTATTCTCTTCAACTCTG	59,0<br>59,0	45<br>42	cgcgtttaaacaagcgagta aggtggag gtctcaacttctcttattgccaac<br>614 tgggttcaaacgcgtttaaacaagcgagtatgttattagcgttatttgcaggtggagatggatacagagttgaagagaataacggttgtttgatgttg 711
LlACT	FP- TGGACGTACTACAGGTATTGTGC<br>RP- ATGGGCACTGTATGGCTCAC	59,0<br>60,0	48<br>55	tggacgtactacaggtattgtgc tggtgatg cactcggtatgtcacgggta<br>433 tatatgccagtggacgtactacaggtattgtgcttgattctggtgatggtgtgagccatacagtgcccat 502

Table S4. (**A**) Comparison of DELLAs derived from *Lupinus luteus* and *Arabidopsis thaliana* using the DiAlign program (Genomatix). For each pairwise alignment, the similarity (relative to the maximum similarity) and the number of identical amino acids (in % of shorter sequence) are given. Maximum values are underlined. The similarity value of 1.000 marks only the two most similar sequences; it does not necessarily mean that these sequences are identical. (**B)** The sub-cellular localization of LlDELLA1, AtGAI, AtRGA, AtRGL1/2/3 predicted by ProtComp v. 9.0 program.

A

	Arabidopsis_thaliana_GAI (532 aa)	Arabidopsis_thaliana_RGA (587 aa)	Arabidopsis_thaliana_RGA-like1 (511 aa)	Arabidopsis_thaliana_RGA-like2 (547 aa)	Arabidopsis_thaliana_RGA-like3 (523 aa)
Lupinus_luteus_DELLA1 (558 aa)	0.831<br>68 %	0.822<br>65 %	0.692<br>61 %	0.660<br>55 %	0.609<br>55 %
Arabidopsis_thaliana_GAI (532 aa)		<u>1.000</u><br><u>81 %</u>	0.680<br>61 %	0.654<br>57 %	0.607<br>56 %
Arabidopsis_thaliana_RGA (587 aa)			0.680<br>61 %	0.681<br>57 %	0.597<br>57 %
Arabidopsis_thaliana_RGA-like1 (511 aa)				0.712<br>63 %	0.659<br>60 %
Arabidopsis_thaliana_RGA-like2 (547 aa)					0.811<br>71 %

B

```
5724 multiple located sequences are accepted
ProtComp Version 9.0. Identifying sub-cellular location (Plant)
Seq name: Lupinus_luteus_DELLA1, Length=558
Significant similarity in Location DB - Nuclear
Database sequence: AC=Q9SLH3 Location:Nuclear  DE  DELLA protein RGA;||3662
Score=90, Sequence length=588, Alignment length=505
Predicted by Neural Nets - Extracellular (Secreted) with score    0.9
Integral Prediction of protein location: Nuclear with score    9.0
Location weights:     LocDB / PotLocDB / Neural Nets / Pentamers / Integral
 Nuclear               10.0 /     3.0 /       0.00 /     0.45 /    9.03
 Plasma membrane        0.0 /     0.0 /       0.94 /     0.00 /    0.61
 Extracellular          0.0 /     0.0 /       0.94 /     0.16 /    0.09
 Cytoplasmic            0.0 /     0.0 /       0.00 /     2.16 /    0.00
 Mitochondrial          0.0 /     0.0 /       0.00 /     1.51 /    0.00
 Endoplasm. retic.      0.0 /     0.0 /       0.00 /     0.49 /    0.00
 Peroxisomal            0.0 /     0.0 /       0.93 /     0.00 /    0.08
 Golgi                  0.0 /     0.0 /       0.19 /     0.24 /    0.00
 Chloroplast            0.0 /     0.0 /       0.00 /     0.21 /    0.00
 Vacuolar               0.0 /     0.0 /       0.00 /     0.05 /    0.20
```

```
5724 multiple located sequences are accepted
ProtComp Version 9.0. Identifying sub-cellular location (Plant)
Seq name: Arabidopsis_thaliana_GAI, Length=532
Significant similarity in Location DB - Nuclear
Database sequence: AC=Q9LQT8 Location:Nuclear  DE  DELLA protein GAI;||3372
Score=99, Sequence length=534, Alignment length=528
Predicted by Neural Nets - Extracellular (Secreted) with score    0.9
Integral Prediction of protein location: Nuclear with score    8.9
Location weights:     LocDB / PotLocDB / Neural Nets / Pentamers / Integral
 Nuclear               10.0 /     3.0 /       0.00 /     0.34 /    8.95
 Plasma membrane        0.0 /     0.0 /       0.94 /     0.05 /    0.62
 Extracellular          0.0 /     0.0 /       0.94 /     0.60 /    0.09
 Cytoplasmic            0.0 /     0.0 /       0.00 /     1.62 /    0.00
 Mitochondrial          0.0 /     0.0 /       0.00 /     2.02 /    0.00
 Endoplasm. retic.      0.0 /     0.0 /       0.00 /     0.29 /    0.00
 Peroxisomal            0.0 /     0.0 /       0.93 /     0.00 /    0.07
 Golgi                  0.0 /     0.0 /       0.19 /     0.24 /    0.00
 Chloroplast            0.0 /     0.0 /       0.00 /     0.07 /    0.02
 Vacuolar               0.0 /     0.0 /       0.00 /     0.00 /    0.25
```

```
5724 multiple located sequences are accepted
ProtComp Version 9.0. Identifying sub-cellular location (Plant)
Seq name: Arabidopsis_thaliana_RGA, Length=587
Significant similarity in Location DB - Nuclear
Database sequence: AC=Q9SLH3 Location:Nuclear  DE  DELLA protein RGA;||3662
Score=100, Sequence length=588, Alignment length=587
Predicted by Neural Nets - Extracellular (Secreted) with score    1.0
Integral Prediction of protein location: Nuclear with score    8.9
Location weights:     LocDB / PotLocDB / Neural Nets / Pentamers / Integral
 Nuclear               10.0 /     3.0 /       0.00 /     0.53 /    8.91
 Plasma membrane        0.0 /     0.0 /       1.00 /     0.06 /    0.74
 Extracellular          0.0 /     0.0 /       1.00 /     0.43 /    0.00
 Cytoplasmic            0.0 /     0.0 /       0.00 /     2.23 /    0.00
 Mitochondrial          0.0 /     0.0 /       0.00 /     1.43 /    0.00
 Endoplasm. retic.      0.0 /     0.0 /       0.00 /     0.26 /    0.00
 Peroxisomal            0.0 /     0.0 /       1.00 /     0.00 /    0.08
 Golgi                  0.0 /     0.0 /       0.00 /     0.12 /    0.00
 Chloroplast            0.0 /     0.0 /       0.00 /     0.11 /    0.00
 Vacuolar               0.0 /     0.0 /       0.00 /     0.06 /    0.27
```

```
5724 multiple located sequences are accepted
ProtComp Version 9.0. Identifying sub-cellular location (Plant)
Seq name: Arabidopsis_thaliana_RGA-like1, Length=511
Significant similarity in Location DB - Nuclear
Database sequence: AC=Q9C8Y3 Location:Nuclear  DE  DELLA protein RGL1;||3663
Score=100, Sequence length=512, Alignment length=511
Predicted by Neural Nets - Extracellular (Secreted) with score    0.9
Integral Prediction of protein location: Nuclear with score    8.8
Location weights:     LocDB / PotLocDB / Neural Nets / Pentamers / Integral
 Nuclear               10.0 /     3.0 /       0.00 /     0.06 /    8.80
 Plasma membrane        0.0 /     0.0 /       0.95 /     0.11 /    0.62
 Extracellular          0.0 /     0.0 /       0.95 /     0.33 /    0.04
 Cytoplasmic            0.0 /     0.0 /       0.00 /     1.25 /    0.00
 Mitochondrial          0.0 /     0.0 /       0.00 /     2.32 /    0.07
 Endoplasm. retic.      0.0 /     0.0 /       0.00 /     0.50 /    0.00
 Peroxisomal            0.0 /     0.0 /       0.95 /     0.00 /    0.07
 Golgi                  0.0 /     0.0 /       0.15 /     0.27 /    0.00
 Chloroplast            0.0 /     0.0 /       0.00 /     0.36 /    0.07
 Vacuolar               0.0 /     0.0 /       0.00 /     0.03 /    0.33

The protein is possibly multilocated: Nucleus_and_Membrane due to SBLAST search in MultiLocDB
***************************************
```

```
5724 multiple located sequences are accepted
ProtComp Version 9.0. Identifying sub-cellular location (Plant)
Seq name: Arabidopsis_thaliana_RGA-like2, Length=547
Significant similarity in Location DB - Nuclear
Database sequence: AC=Q8GXW1 Location:Nuclear  DE  DELLA protein RGL2;||3664
Score=100, Sequence length=548, Alignment length=547
Predicted by Neural Nets - Extracellular (Secreted) with score    1.0
Integral Prediction of protein location: Nuclear with score    8.9
Location weights:     LocDB / PotLocDB / Neural Nets / Pentamers / Integral
 Nuclear               10.0 /     3.0 /       0.00 /     0.15 /    8.94
 Plasma membrane        0.0 /     0.0 /       0.96 /     0.01 /    0.70
 Extracellular          0.0 /     0.0 /       0.96 /     0.43 /    0.00
 Cytoplasmic            0.0 /     0.0 /       0.00 /     1.99 /    0.00
 Mitochondrial          0.0 /     0.0 /       0.00 /     1.98 /    0.00
 Endoplasm. retic.      0.0 /     0.0 /       0.00 /     0.34 /    0.00
 Peroxisomal            0.0 /     0.0 /       0.96 /     0.00 /    0.07
 Golgi                  0.0 /     0.0 /       0.11 /     0.22 /    0.00
 Chloroplast            0.0 /     0.0 /       0.00 /     0.12 /    0.03
 Vacuolar               0.0 /     0.0 /       0.00 /     0.03 /    0.27
```

```
5724 multiple located sequences are accepted
ProtComp Version 9.0. Identifying sub-cellular location (Plant)
Seq name: Arabidopsis_thaliana_RGA-like3, Length=523
Significant similarity in Location DB - Nuclear
Database sequence: AC=Q8GXW1 Location:Nuclear  DE  DELLA protein RGL2;||3664
Score=89, Sequence length=548, Alignment length=468
Predicted by Neural Nets - Extracellular (Secreted) with score    0.9
Integral Prediction of protein location: Nuclear with score    8.9
Location weights:     LocDB / PotLocDB / Neural Nets / Pentamers / Integral
 Nuclear               10.0 /     3.0 /       0.00 /     0.09 /    8.88
 Plasma membrane        0.0 /     0.0 /       0.94 /     0.01 /    0.61
 Extracellular          0.0 /     0.0 /       0.94 /     0.51 /    0.10
 Cytoplasmic            0.0 /     0.0 /       0.00 /     1.60 /    0.00
 Mitochondrial          0.0 /     0.0 /       0.00 /     2.37 /    0.03
 Endoplasm. retic.      0.0 /     0.0 /       0.00 /     0.20 /    0.00
 Peroxisomal            0.0 /     0.0 /       0.93 /     0.00 /    0.07
 Golgi                  0.0 /     0.0 /       0.19 /     0.32 /    0.00
 Chloroplast            0.0 /     0.0 /       0.00 /     0.20 /    0.06
 Vacuolar               0.0 /     0.0 /       0.00 /     0.00 /    0.26
```
